# An RNA interference screen for identifying downstream effectors of the p53 and pRB tumour suppressor pathways involved in senescence

**DOI:** 10.1186/1471-2164-12-355

**Published:** 2011-07-08

**Authors:** Emilie Rovillain, Louise Mansfield, Christopher J Lord, Alan Ashworth, Parmjit S Jat

**Affiliations:** 1 Department of Neurodegenerative Disease, UCL Institute of Neurology, Queen Square, London WC1N 3BG, UK; 2 Breakthrough Breast Cancer Research Centre, The Institute of Cancer Research,237 Fulham Road, London SW3 6JB, UK

**Keywords:** Cellular senescence, RNA interference screen, senescence bypass, conditionally immortal cells

## Abstract

**Background:**

Cellular senescence is an irreversible cell cycle arrest that normal cells undergo in response to progressive shortening of telomeres, changes in telomeric structure, oncogene activation or oxidative stress and acts as an important tumour suppressor mechanism.

**Results:**

To identify the downstream effectors of the p53-p21 and p16-pRB tumour suppressor pathways crucial for mediating entry into senescence, we have carried out a loss-of-function RNA interference screen in conditionally immortalised human fibroblasts that can be induced to rapidly undergo senescence, whereas in primary cultures senescence is stochastic and occurs asynchronously. These cells are immortal but undergo a rapid irreversible arrest upon activation of the p53-p21 and p16-pRB pathways that can be readily bypassed upon their inactivation. The primary screen identified 112 known genes including p53 and another 29 shRNAmirs targetting as yet unidentified loci. Comparison of these known targets with genes known to be up-regulated upon senescence in these cells, by micro-array expression profiling, identified 4 common genes TMEM9B, ATXN10, LAYN and LTBP2/3. Direct silencing of these common genes, using lentiviral shRNAmirs, bypassed senescence in the conditionally immortalised cells.

**Conclusion:**

The senescence bypass screen identified TMEM9B, ATXN10, LAYN and LTBP2/3 as novel downstream effectors of the p53-p21 and p16-pRB tumour suppressor pathways. Although none of them has previously been linked to cellular senescence, TMEM9B has been suggested to be an upstream activator of NF-κB signalling which has been found to have a causal role in promoting senescence. Future studies will focus on determining on how many of the other primary hits also have a casual role in senescence and what is the mechanism of action.

## Background

Normal somatic cells undergo a finite number of divisions before entering a state of irreversible growth arrest termed cellular senescence [1]. This is triggered in response to a variety of intrinsic and extrinsic stimuli including progressive telomere shortening or changes in telomeric structure at the ends of chromosomes or other forms of genotoxic stress such as oncogene activation, or DNA damage or oxidative stress, resulting in a DNA damage response and growth arrest via activation of the p53 tumour suppressor pathway [2,3]. Non-genotoxic stress induces senescence by a telomere independent mechanism involving activation of the p16-pRB pathway by up-regulation of p16^INK4a ^[3,4].

Cellular senescence acts as an important tumour suppressor mechanism. Overcoming senescence and acquiring a limitless replicative potential has been proposed to be one of the key events required for malignant transformation [5]. Senescence is thought to have evolved as an example of antagonistic pleiotrophy, whereby its beneficial traits in a reproductively active individual have deleterious effects later in life [6,7]. The underlying mechanisms that control cellular senescence, the signal transduction pathways involved and how the diverse signals that result in senescence are all integrated, remain poorly defined. Moreover the downstream effectors of the p53-p21 and p16-pRB pathways that result in the irreversible growth arrest and entry into senescence are unknown.

The discovery of RNA interference as a mechanism to silence gene expression has revolutionized studies on mammalian gene expression and has permitted loss-of-function genome-wide genetic screens, to identify genes involved in a variety of cellular processes, to be performed [8-12]. A number of shRNA libraries have been developed for carrying out such genome-wide screens, one of which is the pSM2 Retroviral shRNAmir library [13] (Thermo Scientific Open Biosystems). This library has several unique features that make it very versatile and efficient for large-scale screens particularly the human microRNA-30 (miR30) adapted design which increases knockdown specificity and efficiency [14].

Here we present a RNA interference screen using the human pSM2 retroviral shRNAmir library, carried out in the conditionally immortal HMF3A human fibroblasts, to identify genes whose silencing bypasses senescence arrest induced by activation of the p53-p21 and p16-pRB pathways. The primary screen identified 112 known genes and another 29 shRNAmirs targetting as yet unidentified loci. Comparison of the known targets with genes known to be up-regulated upon senescence by micro-array expression profiling, identified 4 common genes whose expression was reversed when senescence was bypassed upon inactivation of the p53-p21 and p16-pRB pathways.

## Results

**Figure 1:**
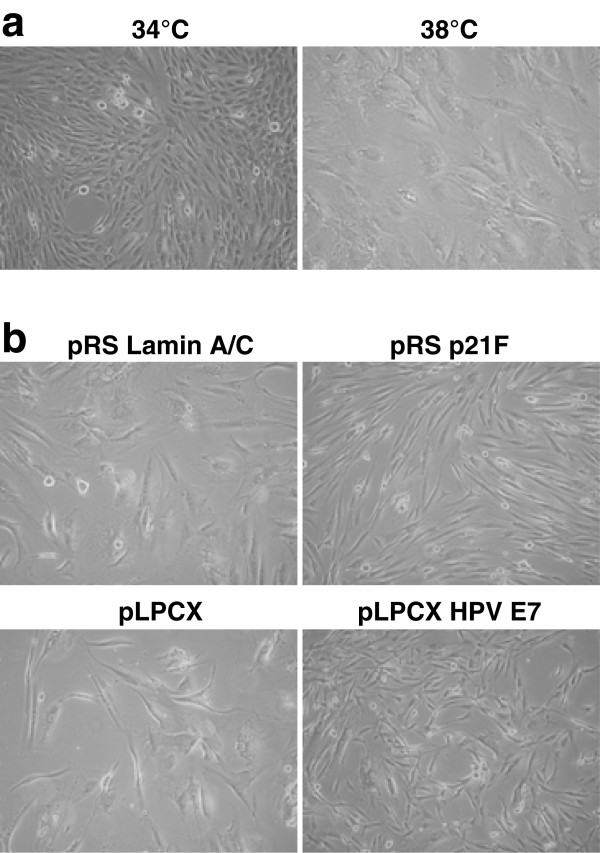
**Characteristics of CL3^EcoR ^cells**. **a**: CL3^EcoR ^cells are immortal at 34°C but undergo a senescence arrest upon shift up 38°C. **b**: Senescence is bypassed upon silencing of p21^CIP1 ^using pRSp21F or sequestration of RB family of proteins by HPV16 E7.

To directly identify the downstream effectors of the p53-p21 and p16-pRB pathways, we have utilized the conditionally immortal HMF3A human fibroblasts that were derived by immortalising adult human mammary fibroblasts with the catalytic subunit of human telomerase and a thermolabile U19tsA58 mutant of SV40 Large T antigen [15]. These cells are immortal if grown at 34°C but undergo a senescence arrest upon inactivation of the thermolabile U19tsA58 T antigen resulting in the activation of the p53-p21 and p16-pRB pathways [15]. They are stringently temperature sensitive but senescence can be readily bypassed by inactivation of the p53-p21 or the p16-pRB pathway [16]. To facilitate efficient transduction of these cells by retroviral infection, they were transduced with the full length murine ecotropic retroviral receptor and CL3^EcoR ^cells derived, that most closely mirror the parental cells [16]. The temperature dependent senescent arrest of CL3^EcoR ^cells and its bypass upon inactivation of p21^CIP1 ^by silencing or sequestration of the RB family of proteins by HPV16 E7 are shown in Figure 1.

**Figure 2:**
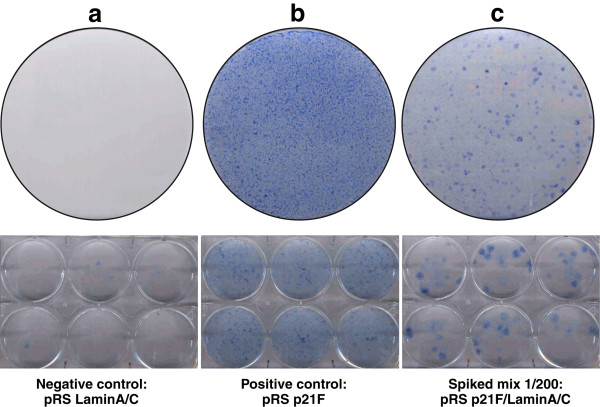
**Sensitivity of the screen**. CL3^EcoR ^cells were infected with retroviruses prepared from pRSLamin A/C **(a)**, pRSp21F **(b)**, or a 1/200 mix of pRSp21F/pRSLamin A/C **(c)**. After puromycin selection, cells were reseeded at 8.5 × 10^4 ^per 15 cm plate or 0.5 × 10^4 ^per well in 6-well plates and shifted to 38°C for 3 weeks.

The pSM2 library version 1.3 comprising 15,148 constructs targetting 9,392 human cancer associated genes was amplified and each 96 well plate used to prepare a pool of plasmid DNA; each of the 100 pools contained between 150 to 200 different shRNAmir constructs with each gene being represented by 1 to 3 shRNAmirs. To ensure that CL3^EcoR ^cells were sufficiently sensitive to identify a single shRNAmir construct in a pool of 200 shRNAmir constructs, pRSp21F (a p21^CIP1 ^shRNA construct) [17] was mixed in a ratio of 1:200 with pRSLaminA/C and used to assay bypass of senescence in CL3^EcoR ^cells. The pRSp21F construct was used because the pSM2 library version 1.3 did not contain any silencing constructs for p21^CIP1^. Silencing of LaminA/C did not bypass senescence, very few growing colonies were obtained (Figure 2a) whereas silencing of p21^CIP1 ^was very efficient and produced essentially a confluent monolayer of growing cells (Figure 2b). The 1:200 p21^CIP1^/LaminA/C mix produced numerous distinct densely growing colonies (Figure 2c) indicating that CL3^EcoR ^cells and the procedure were sufficiently sensitive to generate colonies in which senescence had been overcome.

### ShRNA interference screen

**Figure 3:**
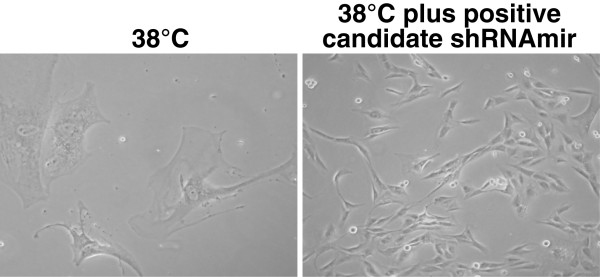
**ShRNA screen**. CL3^EcoR ^cells were transduced with ecotropic retroviruses prepared from each of the 100 pools of shRNAmirs, selected with puromycin, reseeded and shifted up to 38°C for 3 weeks. Densely growing colonies were considered to have bypassed senescence and potentially contain candidate shRNAmirs.

The formula: ln [1-0.95]/ln [1-1/(Library Size)] recommended for genetic screens by the Nolan lab (http://www.stanford.edu/group/nolan/screens/screens.html), suggested that approximately 1000 independent infectious events would be sufficient for a 99% confidence that all shRNAs within a pool had been sampled. To ensure that the screen would be saturating, virus sufficient to yield 10,000 infectious events was utilised for each pool (shown in Additional File 1). The screen was performed in successive waves of 10 pools. To minimise variation and background reversion, CL3^EcoR ^cells were used at the same passage for every pool. Virus prepared from pRSp21F and pRSLamin A/C was used as positive and negative controls respectively to evaluate the level of background and ensure that the complementation assay worked for each round of the screen. Stably transduced cells were trypsinised and reseeded. Three weeks after reseeding, flasks were examined to identify growing colonies; a representative colony is shown in Figure 3. Each colony was examined microscopically to ensure it comprised growing cells and the number of colonies obtained for each pool determined. The number of stably transduced cells, the number of flasks reseeded and the number of colonies obtained for each flask at 38°C are shown in Additional File 2. The flasks which contained more densely growing/bigger colonies (indicated in red in Additional File 2) were trypsinised, replated and used for extracting genomic DNA when confluent.

34 out of 100 pools yielded healthy growing colonies; pools 13, 78 and 82 particularly gave a higher number of colonies which were also larger. Pools 16, 18, 19, 21 and 80 also yielded colonies but they were smaller. For each pool that contained growing colonies, 1 to 4 flasks containing the highest number of growing colonies, were reseeded for extracting genomic DNA and resulted in a total of 81 sub-pools.

### Identification of shRNAmirs

The shRNAmir proviral inserts were by amplified by two rounds of nested PCR using pSM2 specific primers, the amplified products TOPO cloned and plasmid DNA extracted from at least six colonies sequenced to identify all shRNAs present within each pool; for some pools, DNA from many more colonies was sequenced. The hair pin sequence was determined by searching for the miR-30 context and the miR-30 loop that are common to all inserts and frame the hair pin. The sequence of the hair pin was used to identify the target gene by searching the pSM2 data base or by BLASTN analysis of the NCBI human genome data base (http://blast.ncbi.nlm.nih.gov/Blast.cgi). Sequences that could not be linked back to the list of hair pin sequences within the Open Biosystems collection or were not 100% homologous to a gene within Genbank were not pursued and are presented in Additional File 3.

The rescued shRNAmir hair pins identified 112 known genes and another 29 shRNAmirs targetting as yet unidentified loci. For each pool, the number of times that sequence was obtained, the corresponding insert reference, gene name and the number of shRNAmir constructs for that gene within the SM2 library are shown in Table 1. The last column of the table indicates if the recovered insert was a match to a hair pin present in that particular pool ("match") or if it was present within another pool ("listed in pool X"). 14 hair pins were isolated from incorrect pools. Some inserts were detected several times in multiple pools. For example, the hair pin v2HS_119967 (corresponding to LOC155004) listed within pool 52 was also identified from pool 3, 4, 5, 9, 12, 30, 59, 60, 64, 71, 72, 74, 77, and 98 suggesting that there may have been some cross-contamination of the library perhaps during replica plating. However, this was not a problem since the aim of our screen was to identify shRNAmirs that are able to bypass senescence and does not depend upon the identity of the pool. Since this insert was isolated from so many pools, it could be that it is a strong positive or that it was highly represented within the library and likely to be a false positive.

**Table 1:** Primary screen

Pool	Insert	Gene name	Gene symbol	Freq	Cons per Gene	Library location
**3**	v2HS_63142	keratin associated protein 5-9	KRTAP5-9	3/9	1	match
	v2HS_119967	LOC155004	LOC155004	4/9	2	pool 52
	v2HS_56766	acyl-CoA synthetase medium-chain family member 3	ACSM3	1/9	2	match
	v2HS_59294	glycoprotein hormone alpha 2	GPHA2	1/9	1	match
	
	v2HS_53974	PRO0255 protein	PRO0255	5/8	1	match
	v2HS_63482	paired box gene 1	PAX1	2/8	1	match

**4**	v2HS_119967	LOC155004	LOC155004	2/10	2	pool 52
	v2HS_66751	LOC344082	LOC344082	4/10	1	match
	v2HS_70011	serum amyloid A-like 1	SAAL1	2/10	2	match
	v2HS_98079	human solute carrier family 22	SLC22A3	1/10	2	pool 79

**5**	v2HS_108647	LOC349868	LOC349868	3/8	1	pool 79
	v2HS_70473	polymerase (DNA directed), mu	POLM	2/8		
	v2HS_71958	human olfactory receptor, family 5, subfamily P, member 3	OR5P3	3/8	1	match
	
	v2HS_119967	LOC155004	LOC155004	7/12	2	pool 52
	v2HS_66652	protein phosphatase 3, catalytic subunit, β isoform	PPP3CB	1/12	1	match
	v2HS_70473	polymerase (DNA directed), mu	POLM	4/12		

**7**	v2HS_112910	human cyclin-dependent kinase 8	CDK8	1/10	3	match
	v2HS_98079	solute carrier family 22, member3	SLC22A3	1/10	2	pool 79
	v2HS_97017	sterile alpha motif containing 4a	SAMD4A	1/10		
	
	v2HS_69776	cytochrome P450, family 4, subfamily Z, polypeptide	CYP4Z2P	1/10	2	match
		2 pseudogene				

**9**	v2HS_108647	LOC349868	LOC349868	1/8	1	pool 79
	v2HS_119967	LOC155004	LOC155004	1/8	2	pool 52
	v2HS_62506	family with sequence similarity 181, member B	FAM181B	1/8	1	match
	
	v2HS_55950	prostate stem cell antigen	PSCA	2/13	1	match
	v2HS_119967	LOC155004	LOC155004	3/13	2	pool 52
	v2HS_70312	TRK-fused gene	TFG	4/13	3	match
	v2HS_71740	ataxin 10	ATXN10	1/13	1	match
	v2HS_98079	solute carrier family 22, member 3	SLC22A3	1/13	2	pool 79
	v2HS_65121	sorting nexin 12	SNX12	1/13	1	match
	
	v2HS_64384	amyloid beta precursor protein binding family A member 2	APBA2	1/6	1	match
	v2HS_97017	sterile alpha motif containing 4A	SAMD4A	1/6		

**12**	v2HS_119967	LOC155004	LOC155004	5/11	2	pool 52
	v2HS_58950	signal-regulatory protein beta 2	SIRPB2	3/11	2	match
	
		protein tyrosine phosphatase, non-receptor type 13	PTPN13	1/10		
	v2HS_119967	LOC155004	LOC155004	5/10	2	pool 52

**13**	v2HS_55731	phenylalanine-tRNA synthetase-like, beta subunit	FARSLB	1/12	2	match
	v2HS_59258	dynein, light chain, roadblock-type 1	DYNLRB1	3/12	1	match
	v2HS_59891	TAO kinase 1	TAOK1	5/12	3	match
	v2HS_162164	iodotyrosine deiodinase	IYD	1/12		
		cell cycle exit and neuronal differentiation 1	CEND1	1/12		
	
		transmembrane protein 168	TMEM168	2/5		
	v2HS_68714	abl-interactor 1	ABL1	3/5	3	match
	
		cell cycle exit and neuronal differentiation 1	CEND1	4/6		
	v2HS_58952	signal regulatory protein gamma	SIRPG	1/6	2	pool 14
	v2HS_59653	LOC342404	LOC342404	1/6	1	match
	
	v2HS_55731	phenylalanyl-tRNA synthetase like, beta subunit	FARSLB	1/15	2	match
	v2HS_64320	Smith-Magenis syndrome chromosome region, candidate 7	SMCR7	1/15	1	match
	v2HS_71174	peroxisome proliferator-activated receptor, gamma	PPARGC1A	3/15	1	match
	v2HS_71453	similar to IAP-associated factor VIAF1; phosducin-like		2/15	1	match

**16**	v2HS_68478	CD28 antigen	CD28	8/12	2	pool 13
	v2HS_63107	dopamine beta hydoxylase	DBH	2/12	1	match
	
	v2HS_61750	LOC100129230	LOC100129230	7/13	3	match
		LOC9142	LOC9142	2/13		
	V2HS_62831	membrane bound O-acyltransferase domain containing 1	MBOAT1	3/13	2	match

**18**	v2HS_63989	STAR-related lipid transfer (START) domain containing 6	STARD6	1/14	2	match
		LOC730256	LOC730256	1/14		

**19**	v2HS_58958	TMEM9 domain family, member B/C11orf15	TMEM9B	3/10	1	match
	v2HS_57051	RNA binding motif, single stranded interacting protein 1	RBMS1	5/10	1	match
	v2HS_106158	LOC345672	LOC345672	1/10	2	pool 82
	
	v2HS_59560	chromosome 13 open reading frame 15	C13orf15	10/10	1	match

**21**	v2HS_55312	glucosamine-phosphate N-acetyltransferase 1	GNPNAT1	6/9	2	match
	v2HS_68437	LOC157503	LOC157503	2/9	3	match

**30**	v2HS_119967	LOC155004	LOC155004	6/14	2	pool 52
	v2HS_34338	protein phosphatase 4, regulatory subunit 2	PPP4R2	7/14	3	match
	
	v2HS_119967	LOC155004	LOC155004	1/13	2	pool 52
	v2HS_36467	solute carrier family 33 (acetyl-CoA transporter), member 1	SLC33A1	8/13	3	match
	v2HS_46793	ubiquitin-like modifier activating enzyme 3	UBA3	1/13	1	match

**32**	v2HS_42104	citrate lyase beta like (CLYBL), transcript variant 1	CLYBL	5/7	1	match
		LOC100129563	LOC 10012956	1/7		

**41**	v2HS_48278	choline kinase-like	CHKL	6/6	1	match

**54**	v2HS_112629	basic transcription factor 3, like 1	BTF3L1	1/5	1	match
	v2HS_121153	LOC221399	LOC221399	2/5	1	match
	v2HS_125538	LOC350103	LOC350103	1/5	1	match
	
	v2HS_129527	LOC351851	LOC351851	6/6	1	match
	
	v2HS_112629	basic transcription factor 3, like 1	BTF3L1	2/6	1	match

**55**	v2HS_117465	SH3 domain binding glutamic acid-rich protein like 2	SH3BGRL2	2/6	1	match
	v2HS_119051	LOC90841	LOC90841	1/6	1	match
	
	v2HS_116174	YTH domain containing 2	YTHDC2	1/7	2	pool 58
	v2HS_119120	hypothetical protein FLJ20032	FLJ20032	6/7	2	match

**56**	V2HS_120429	six-twelve leukemia (STL), non-coding RNA	STL	5/6	1	match
	
	v2HS_115231	rab23, member RAS oncogene family	RAB23	4/6	4	match
	v2HS_117914	transketolase-like 2	TKTL2	2/6	1	match
	
	v2HS_117239	chromosome 9 open reading frame 58	C9orf58	3/6	1	match
	v2HS_120429	six-twelve leukemia (STL), non-coding RNA	STL	1/6	1	match
	v2HS_119206	lon peptidase 2, peroxisomal	LONP2	2/6	1	pool 146

**58**	v2HS_116174	YTH domain containing 2	YTHDC2	1/4	2	match
	v2HS_118722	layilin	LAYN	2/4	2	match
	
	v2HS_112838	ectonucleoside triphosphate diphosphohydrolase 3	ENTPD3	3/7	4	match
	v2HS_112982	chromodomain helicase DNA binding protein 3	CHD3	1/7	3	match
	v2HS_116174	YTH domain containing 2	YTHDC2	1/7	2	match
	
	v2HS_112838	ectonucleoside triphosphate diphosphohydrolase 3	ENTPD3	1/12	4	match
	v2HS_118254	WD repeat and FYVE domain containing 2	WDFY2	1/12	1	match
	v2HS_122548	similar to ribosomal protein S3A	RPS3A	4/12	1	pool 53
	v2HS_120982	coiled coil domain containing 129	CCDC129	5/12	2	match

**59**	v2HS_111554	interleukin 2	IL2	8/10	2	match
	v2HS_115544	DEAD (Asp-Glu-Ala-Asp) box polypeptide 47	DDX47	2/10	4	match
	
	v2HS_116377	transmemembrane protein 135	TMEM135	1/9	2	match
	v2HS_117064	CD1E molecule	CD1E	3/9	1	match
	v2HS_119967	LOC155004	LOC155004	4/9	2	pool 52
	v2HS_120757	olfactory receptor, family 8, subfamily K, member 1	OR8K1	1/9	2	match
	
	v2HS_108647	LOC349868	LOC349868	1/8	1	pool 79
	v2HS_115544	DEAD (Asp-Glu-Ala-Asp) box polypeptide 47	DDX47	3/8	4	match
	v2HS_116833	intermediate filament protein syncoilin	SYNC	1/8	2	match

**60**	v2HS_121585	dual specificity phosphatase 3	DUSP3	10/11	1	match
	
	v2HS_117903	glutamine rich 2	QRICH2	4/11	1	match
	v2HS_119967	LOC155004	LOC155004	1/11	2	pool 52
	v2HS_121585	dual specificity phosphatase 3	DUSP3	2/11	1	match
	v2HS_128131	LOC351061	LOC351061	2/11	1	match
	v2HS_98079	solute carrier family 22 member 3	SLC22A3	1/11	2	pool 79
	
	v2HS_119967	LOC155004	LOC155004	8/10	2	pool 52
	v2HS_121585	dual specificity phosphatase 3	DUSP3	2/10	1	match

**64**	v2HS_119967	LOC155004	LOC155004	10/11	2	pool 52
	
	v2HS_121013	protease serine 54	PRSS54	3/12	1	match

**66**	v2HS_117675	latent transforming growth factor β binding protein 2/3	LTBP2/3	15/15		
	
	v2HS_118530	RNA exonuclease 1 homolog like 1	REXO1L1	5/13	3	match
	v2HS_117675	latent transforming growth factor β binding protein 2/3	LTBP2/3	8/13		

	v2HS_118530	REX1, RNA exonuclease 1 homolog-like 2	REXO1L1	1/11	3	match

**71**	v2HS_103818	LOC284804	LOC284804	2/16	1	pool 78
	v2HS_119967	LOC155004	LOC155004	9/16	2	pool 52
	v2HS_97981	LOC51152	LOC51152	5/16	2	pool 79

**72**	v2HS_119967	LOC155004	LOC155004	9/10	2	pool 52

**74**	v2HS_119967	LOC155004	LOC155004	11/12	2	pool 52

**77**	v2HS_119967	LOC155004	LOC155004	12/12	2	pool 52

**78**	v2HS_94763	paired related homeobox 1	PRRX1	1/8	1	match
	v2HS_99138	solute carrier family 25	SLC25A21	7/8	2	match
	
	v2HS_102207	transmembrane protein 63B	TMEM63B	1/12	1	match
	v2HS_103818	LOC284804	LOC284804	4/12	1	match
	V2HS_95356	armadillo repeat containing, X-linked 2	ARMCX2	2/12	2	match
	v2HS_98650	mitochondrial ribosomal protein 63	MRP63	1/12	1	match
	
	v2HS_102155	similar to hypothetical protein KIAA0286 (HA6800)	KIAA0286	1/8	1	match
	v2HS_108506	LOC349839	LOC349839	1/8	2	match
	
	v2HS_96236	zinc finger and BTB domain containing 1	ZBTB1	2/8	1	match
	v2HS_184999	eukaryotic initiation factor 4A isoform 1	EIF4A1	3/10		
	v2HS_96236	zinc finger and BTB domain containing 1	ZBTB1	1/10	1	match

**79**	v2HS_105974	LOC345597	LOC345597	3/11	1	match
	v2HS_184999	eukaryotic initiation factor 4A isoform 1	EIF4A1	3/11		
	v2HS_98079	solute carrier family 22, member 3	SLC22A3	4/11	2	match
	
	v2HS_108647	LOC349868	LOC349868	2/7	1	match
	v2HS_98079	solute carrier family 22, member 3	SLC22A3	5/7	2	match
	
	v2HS_101943	anthrax toxin receptor-like	ANTXRL	1/11	3	match
	v2HS_105093	human solute carrier family 35, member F4	SLC35F4	2/11	1	match
	v2HS_106472	LOC345713	LOC345713	1/11	1	match
	v2HS_107395	LOC346589	LOC346589	1/11	1	match
	v2HS_91777	nuclear receptor co-repressor N-Cor 1	NCOR1	4/11	2	match
		Non-protein coding RNA 282	NCRNA00282	1/11		

**80**	v2HS_102441	adenylate cyclase 1	ADCY1	5/11	1	match
	v2HS_130882	glutamate receptor, metabotropic 3	GRM3	1/11	3	pool 91
	v2HS_97368	yippee-like 5	YPEL5	2/11	1	match
	
	v2HS_102441	adenylate cyclase 1	ADCY1	5/10	1	match

**82**	v2HS_184999	eukaryotic initiation factor 4A isoform 1	EIF4A1	1/11		
	v2HS_101845	human prickle-like 2 (Drosophila)	PRICKLE2	3/11	1	match
	v2HS_109096	LOC349975	LOC349975	1/11	1	pool 84
	v2HS_99423	coiled coil domain containing 70	CCDC70	1/11	1	match
	
	v2HS_109596			1/14		
	v2HS_108399	LOC349811	LOC349811	3/14	1	match
	v2HS_184999	eukaryotic initiation factor 4A isoform 1	EIF4A1	1/14		
	v2HS_93536	proteolipid protein 1	PLP1	5/14	1	match
	v2HS_97017	sterile alpha motif domain containing 4A	SAMD4A	1/14		
	
	v2HS_106158	LOC345672	LOC345672	3/14	2	match
	v2HS_93615	p53	TP53	3/14	1	match
	v2HS_95112	RAS p21 protein activator 4	RASA4	1/14	1	match
	v2HS_99525	BCL2 like 12	BCL2L12	1/14	2	match
	v2HS_97017	sterile alpha motif domain containing 4A	SAMD4A	4/14		
	
	v2HS_94640	aryl hydrocarbon receptor nuclear translocator-like	ARNTL	3/10	2	match
	v2HS_100174	desmoglein 4	DSG4	2/10	2	pool 145
	v2HS_96026	adnp homeobox 2	ADNP2	3/10	1	match
	v2HS_100819	Rho GTPase activating protein 20	ARHGAP20	2/10	1	match

**84**	v2HS_106409	LOC345700	LOC345700	1/11	1	match
	v2HS_95019	zinc finger protein 16	ZNF16	9/11	1	match
	v2HS_97152	ubiquitin-conjugating enzyme E2, J1	UBE2J1	1/11	1	match
	
	v2HS_95019	zinc finger protein 16	ZNF16	10/11	1	match

**94**	v2HS_141495	zinc finger protein 454	ZNF454	7/12	2	pool 96

**95**	v2HS_130457	radial spoke head 10 homolog B (Chlamydomonas)	RSPH10B	6/7	3	match
	v2HS_131154	potassium inwardly-rectifying channel, subfamily J	KCNJ2	1/7	3	match
	
	v2HS_184999	eukaryotic initiation factor 4A isoform 1	EIF4A1	1/3		

**96**		chromosome 9 open reading frame 123	C9orf123	5/13		

**98**	v2HS_141367	cDNA FLJ37626/LOC285500	FLJ37626	10/10	2	match
	
	v2HS_119967	LOC155004	LOC155004	2/11	2	pool 52
	v2HS_133299	insulin-like growth factor binding protein 6	IGFBP6	3/11	2	match
	v2HS_135564	chromosome X open reading frame 57	CXORF57	5/11	1	match

For each candidate the DNA pool from which it was isolated, the insert reference, target gene, frequency of isolation, as well as the number of shRNAmir constructs for that gene within the SM2 library are indicated. The last column shows if the recovered insert was a match to a hair pin in that particular pool ("match") or if it was from another pool ("pool X").

Pool 13, 78 and 82 that produced more colonies and colonies that were larger and healthier than others, identified the following genes: Pool 13: DYNLRB1, FARSLB, PPARGC1A, TAOK1, CEND1, ABL1, LOC342404, TMEM168, SIRPG, IYD and SMCR7; Pool 78: PRRX1, SLC25A21, ARMCX2, LOC284804, LOC349839, MRP63, TMEM63B, ZBTB1, KIA0286 and EIF4A1; and Pool 82: EIF4A1, CCDC70, PRICKLE2, PLP1, SAMD4A, RASA4, TP53, ARNTL, BCL2L12, ADNP2, DSG4, LOC345672, LOC349975, LOC349811 and ARHGAP20. Moreover, pool 82, one of the pools that gave the best senescence bypass, contained the only shRNAmir targetting p53, v2HS_93615 identified in the screen, thereby internally validating it.

### Overlap of the primary candidates of the shRNA screen with microarray data for genes up-regulated upon senescence in CL3^EcoR ^cells

To prioritise the candidates identified from the primary screen for functional validation, they were compared to genes up-regulated upon senescence and whose expression was reversed when senescence was abrogated upon inactivation of the p53 and pRB pathways [[16]; microarray expression profiling data is available from Gene Expression Omnibus database accession number GSE24810]. This identified 4 common genes, ATXN10, LAYN, LTBP2/3 and TMEM9B. The microarray expression profiling data presented in Table 2 showed that they were all up-regulated upon senescence growth arrest: ATXN10 by 1.3 fold (p-value 1 × 10^-3^), LAYN by 2 fold (p-value 2 × 10^-4^), LTBP2/3 by 2.5 (p-value 9 × 10^-8^), 1.7 (p-value 5 × 10^-9^), 1.5 (p-value 8 × 10^-4^) and 1.4 fold (p-value 5 × 10^-5^) and TMEM9B by 1.4 (p-value 1 × 10^-7^) and 1.3 fold (p-value 2 × 10^-4^) respectively. The data in Table 2 further show that up-regulation of these candidates was reversed when senescence was bypassed upon inactivation of the p53-p21 and p16-pRB pathways by silencing p53 (pRS_p53) or p21^CIP1 ^(pRS_p21) or by sequestration of the RB family of proteins by HPV16 E7 or by expression of the dominant negative E2F-DB protein.

**Table 2:** Microarray expression profiling data for common genes

Probe	Symbol	GA	p-value	HS	p-value	pRS_p53	p-value	pRS_p21	p-value	E7	p-value	E2F_DB	p-value
208832_at	ATXN10	0.36	1E-03	0.25	3E-02	-0.18	2E-01	-0.35	2E-03	0.02	9E-01	-0.23	4E-02

228080_at	LAYN	1.04	2E-04	0.30	3E-01	-0.95	1E-03	-0.70	2E-02	-0.56	6E-02	-0.11	8E-01

219922_s_at	LTBP2/3	1.32	9E-08	0.66	5E-03	-1.04	2E-05	-1.32	3E-07	-1.19	1E-06	-1.41	4E-08

223690_at	LTBP2/3	0.75	5E-09	-0.14	3E-01	-0.52	1E-05	-0.91	2E-10	-1.27	1E-14	-1.22	3E-14

204682_at	LTBP2/3	0.57	8E-04	-0.48	8E-03	-0.39	4E-02	-0.94	1E-06	-1.62	3E-12	-2.02	4E-15

227308_x_at	LTBP2/3	0.46	5E-05	0.03	9E-01	-0.43	3E-04	-0.57	4E-06	-0.28	2E-02	-0.55	4E-06

218065_s_at	TMEM9B	0.44	1E-07	-0.11	2E-01	-0.33	6E-05	-0.38	9E-06	-0.20	1E-02	-0.18	2E-02

222507_s_at	TMEM9B	0.39	2E-04	-0.19	1E-01	-0.28	1E-02	-0.34	2E-03	-0.12	3E-01	-0.07	6E-01

Log_2 _fold changes in gene expression in CL3^EcoR ^cells upon senescence arrest are indicated as GA and upon shift up of control temperature insensitive HMF3S cells, from 34°C to 38°C, as HS respectively. Also indicated are the data obtained for these genes from CL3^EcoR ^cells in which senescence had been bypassed by silencing of p53 (pRS_p53) or p21^CIP1 ^(pRS_p21) or by inactivation of pRB using HPV16 E7 (E7) or by expression of the dominant negative E2F-DB protein.

**Figure 4:**
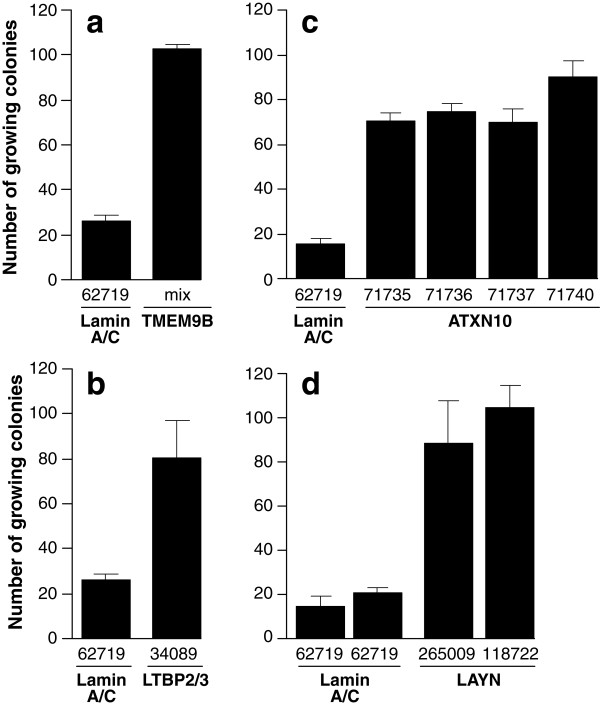
**Silencing targets identified from the primary screen**. CL3^EcoR ^cells were infected in triplicate with lentiviruses prepared from the indicated GIPZ shRNAmir constructs targetting TMEM9B **(a)**, LTBP2/3 **(b)**, ATXN10 **(c) **and LAYN **(d)**. Cells resistant to 6 μg/ml puromycin were isolated, reseeded and assayed for bypass of senescence by culturing at 38°C for 3 weeks. Lentiviruses prepared from the GIPZ shRNAmir Lamin A/C (v2LHS_62719) construct were used as the negative control. For TMEM9B, the mix of constructs comprised v2LHS_247318, 58957, 58958 and 58959.

The identification of TMEM9B was particularly remarkable because the microarray analysis has suggested that senescence growth arrest in CL3^EcoR ^cells is associated with activation of the NF-κB signalling pathway and TMEM9B has previously been shown to be able to activate NF-κB dependent reporter constructs [18,19]. To determine if silencing of TMEM9B would bypass senescence, 4 GIPZ lentiviral silencing constructs (v2LHS_247318, 58957, 58958 and 58959; Thermo Scientific Open Biosystems) targetting TMEM9B were obtained, pooled and introduced into CL3^EcoR ^cells after packaging as lentiviruses. The GIPZ lentiviral library contains the same hair pins as the retroviral library but is more stable and the constructs are packaged as lentiviruses rather than retroviruses. Lentiviral human GIPZ Lamin A/C shRNAmir (v2LHS_62719) was used as a negative control. Silencing of TMEM9B was clearly able to bypass senescence (Figure 4a). Moreover each of the constructs was individually able to overcome senescence arrest with v2LHS_58957 being the most efficient [16].

To determine if ATXN10, LAYN and LTBP2/3 were also able to directly bypass senescence, lentivirus constructs from the GIPZ lentiviral shRNAmir library were used. The complementation assay for LTBP2/3, in Figure 4b, showed that silencing LTBP2/3 with the one available silencing construct (v2LHS_34089) clearly yielded healthy growing colonies. Silencing of ATXN10 was tested using 4 different silencing constructs v2LHS_71735, 71736, 71737 and 71740. All four constructs were able to overcome senescence and yielded more growing colonies than the Lamin A/C negative control (Figure 4c). Silencing of LAYN was tested using 2 different silencing constructs v2LHS_265009 and 118722; both constructs were able to bypass growth arrest and produced growing colonies (Figure 4d).

Taken together our results showed that silencing of TMEM9B, ATXN10, LAYN and LTBP2/3 was able to bypass senescence in the conditionally immortal human fibroblasts.

## Discussion

To directly identify the downstream effectors of the p53-p21 and p16-pRB pathways crucial for mediating entry into senescence, we carried out a loss-of-function RNA interference screen in the conditionally immortal HMF3A human fibroblasts. This identified 112 known genes including p53 and another 29 shRNAmirs targetting unidentified loci. Comparison of these known targets with genes up-regulated upon senescence in these cells identified 4 common genes TMEM9B, ATXN10, LAYN and LTBP2/3. Direct silencing of these common genes using lentiviral shRNAmirs bypasses senescence in the HMF3A cells. Although none of these genes has previously been linked to cellular senescence, TMEM9B has been suggested to be an upstream activator of NF-κB signalling which we have found to have a causal role in promoting senescence.

The 112 known targets identified by the shRNA screen comprise a wide variety of genes but most importantly one of them was the only p53 shRNAmir (v2HS_93615 from pool 82) present within this library, thereby internally validating the screen. Moreover all of the primary targets were identified from single shRNAmirs even though we have subsequently shown that other shRNAmirs corresponding to these targets present within the library are able to bypass senescence. It is not clear why other shRNAmirs were not isolated in the screen; however this is exactly what has been observed previously by others such as Westbrook and colleagues [20]. Nevertheless it remains to be demonstrated which of the targets identified by the primary screen are able to bypass senescence when assayed individually.

TMEM9B was one of the 4 genes in common between the shRNA screen and genes known to be up-regulated upon senescence in HMF3A cells [16]. Moreover expression of TMEM9B was down-regulated when senescence was bypassed upon abrogation of the p53-p21 or p16-pRB pathways. TMEM9B is a glycosylated protein that localises to lysosome membranes and partially to early endosomes. It has been shown to be a component of TNF signalling and a module shared between the interleukin-1 and Toll-like receptor pathways. It is also essential for TNF activation of both NF-κB and MAPK pathways by acting downstream of RIP1 and upstream of the MAPK and IκB kinases at the level of the TAK1 complex [19]. TMEM9B was also identified in a large scale study to identify genes activating NF-κB and MAPK signalling pathways [18]. These results are all consistent with our finding that in the conditionally immortal HMF3A cells, senescence growth arrest is associated with an activation of NF-κB signalling and suppression of this pathway bypasses senescence [16].

The latent TGFβ-binding protein 2/3 (LTBP2/3) hair pin sequence was identified from pool 66. Up-regulation of LTBP2/3 expression upon growth arrest was reversed when senescence was overcome. LTBPs are secreted proteins initially identified through their binding to TGFβ and may be involved in their assembly, secretion and targetting [21]. LTBP2/3 in particular has been found to play an essential role in the secretion and targetting of TGFβ1 [22]. Since silencing of LTBP2/3 can bypass senescence in HMF3A cells, it suggests that LTBP2/3 may be linked with the control of cell growth and be playing a role in suppressing tumour progression perhaps through regulation of TGFβ. This is in accordance with the identification of TGFβ as a senescence-inducing factor in the human lung A549 adenocarcinoma cells [23]. It is also in accordance with several other reports suggesting that TGFβ1 is capable of inducing cellular senescence. For instance, stimulation of human diploid fibroblasts with TGFβ1 triggers the appearance of senescence associated-β-galactosidase activity and increases steady state mRNA levels of senescence associated genes including APO J, fibronectin, and M22 [24-26]; both APO J (clusterin) and fibronectin are up-regulated in CL3 cells upon senescence arrest and this is reversed when senescence is bypassed [16].

Ataxin (ATXN) 10 was slightly up-regulated upon senescence arrest which was reversed upon silencing of p53 and p21^CIP1 ^or ectopic expression of the dominant negative E2F-DB protein. Spinocerebellar ataxia type 10 (SCA10) is a dominantly inherited disorder characterized by ataxia, seizures and anticipation caused by an intronic ATTCT pentanucleotide repeat expansion. The product of SCA10 encodes the novel protein, ATXN10, previously known as E46L, which is widely expressed in the brain and belongs to the family of armadillo repeat proteins. Although clinical features of the disease are well characterized, very little is known about ATXN10. ATXN10 knock down by RNAi has recently been shown to cause increased apoptosis in primary cerebellar cultures, implicated in SCA10 pathogenesis [27,28]. This is in contrast to our finding that silencing of ATXN10 in HMF3A cells by four different shRNAmirs did not cause apoptosis but promoted growth and permitted a bypass of senescence.

Layilin (LAYN) identified from pool 58 was 2 fold up-regulated upon senescence arrest, which was reversed upon abrogation of the growth arrest by inactivation of either the p53-p21 or the p16-pRB pathways. Moreover two different LAYN shRNAmirs were found to directly bypass senescence in HMF3A cells. Layilin is a widely expressed integral membrane hyaluronan receptor, originally identified as a binding partner of talin located in membrane ruffles. Talin is responsible, along with its adaptor proteins, for maintaining the cytoskeleton-membrane linkage by binding to integral membrane proteins and to the cytoskeleton. Recently layilin has been suggested to play a crucial role in lymphatic metastasis of lung carcinoma A549 cells [29].

In addition to the genes described above, a number of other interesting genes particularly TAOK1, RAS4A and ARMCX2 were identified. TAOK1 is a micro-tubule affinity-regulating kinase required for both chromosome congression and checkpoint-induced anaphase delay [30]. It is known to activate the p38MAPK pathway through the specific phosphorylation of MKK3. This is a complex pathway responsive to stress stimuli and involved in cell differentiation and apoptosis and has been shown to have an important causative role in senescence [31]. RAS4A encodes a member of the GAP1 family of GTPase-activating proteins that have been identified to suppress the Ras/mitogen-activated protein kinase pathway in response to an elevation of Ca^2+ ^ions. Stimuli that increase intracellular Ca^2+ ^levels result in the translocation of this protein to the plasma membrane, where it activates Ras GTPase activity resulting in Ras being converted from the active GTP-bound state to the inactive GDP-bound state and suppression of downstream signalling [32]. ARMCX2 encodes a member of the ALEX family of proteins and may play a role in tumour suppression. This protein contains a potential N-terminal transmembrane domain and a single Armadillo repeat; armadillo repeat containing proteins are involved in development, maintenance of tissue integrity and suppressing carcinomas [33].

## Conclusions

The RNA interference screen has identified 112 known candidate proteins including p53 and another 29 shRNAmirs targetting as yet unidentified loci. Although none of them except p53 had previously been linked to senescence or known to be downstream effectors of the p53-p21 and p16-pRB tumour suppressor pathways, directly silencing four of these candidates, TMEM9B, ATXN10, LAYN and LTBP2/3 bypassed senescence in CL3^EcoR ^cells. It remains to be determined whether direct silencing of any of the other primary candidates will also bypass senescence. Any genes that can bypass senescence upon silencing are novel starting points for identifying the signalling networks that act downstream of p53 and pRB to induce cellular senescence. The genes/proteins identified in the screen are also potential tumour suppressors, and a mechanistic dissection of their mode of action and role in cancer will undoubtedly provide new avenues for further research.

## Methods

### Cell Culture

CL3^EcoR ^cells were maintained at 34°C ± 0.5°C [15]. Temperature shift experiments were performed at 38°C ± 0.5°C. Phoenix ecotropic and HEK293T cells were obtained from the ATCC and maintained at 37°C. Cells were grown in Dulbecco's modified Eagles medium (DMEM) supplemented with 10% (v/v) heat inactivated foetal bovine serum, 2 mM glutamine, 100 units/ml penicillin and 100 μg/ml streptomycin. All media and components were obtained from Invitrogen.

### Viral packaging and infection

Lentiviruses were prepared according to Besnier *et al. *[34]. Ecotropic viruses were prepared by transfecting 10 μg of retroviral plasmid DNA into phoenix ecotropic cells by FuGENE 6 Transfection reagent (Roche), according to the manufacturer's instructions. 24 hrs post-transfection, media was changed and fresh medium added. 48 hrs post-transfection, retroviral supernatant was harvested, filtered through a 0.45 μm filter and either used immediately or frozen at -80°C. A second harvest was prepared by adding fresh media to the plates and harvesting the virus supernatant the next day.

Cells were infected with virus supernatants for 24 hrs at 34°C. Four days post-infection, antibiotic selection was added (2 μg/ml puromycin for pRS and pSM2 retroviruses or 6 μg/ml puromycin for pGIPZ shRNAmir lentiviruses; Invitrogen). Selection of cells infected with human GIPZ lentiviral shRNAmir constructs in puromycin at 6 μg/ml, enriches for cells with higher levels of shRNAmir expression. For the senescence bypass assay, the stably transduced cells were plated at 5 × 10^4 ^cells in T-75 flasks or at 1.6 × 10^4 ^cells in T-25 flasks and incubated overnight at 34°C. Next day the medium was changed and the cells shifted to 38°C for 3 weeks. Flasks which contained more densely growing or bigger colonies were trypsinised, replated and used for extracting genomic DNA when confluent.

## Competing interests

The authors declare that they have no competing interests.

## Authors' contributions

ER and LM-carried out the screen. ER and PSJ rescued the inserts. CJL and AA amplified and provided the shRNAmir library. PSJ wrote the manuscript. All authors have read and approved the final manuscript.

## Supplementary Material

Additional file 1 **Volume of virus supernatants used for the screen**. The table shows the volume of virus supernatants used for each of the pools. Click here for file

Additional file 2 **Primary screen**. This table shows the pool number, the number of cells obtained after puromycin selection, the number of T-75 and T-25cm2 flasks reseeded and the number of growing colonies observed after 3 weeks at 38°C. Numbers indicated in red correspond to flasks that were reseeded for extracting genomic DNA. Click here for file

Additional file 3 **Unidentified inserts**. This table shows the hair pin sequences that were not 100% homologous to a gene by BLASTN analysis of the NCBI human genome data base (http://blast.ncbi.nlm.nih.gov/Blast.cgi) or could not be linked back to a hair pin sequence within the Open Biosystems SM2 library; the number of times the insert was isolated is also indicated. Click here for file
